# Supplementary figures and images for: Taphonomy and taxonomy of a juvenile lambeosaurine (Ornithischia: Hadrosauridae) bonebed from the late Campanian Wapiti Formation of northwestern Alberta, Canada
Source: PeerJ. 2021 May 4;9:e11290. doi: 10.7717/peerj.11290 (PMC8103918; doi:10.7717/peerj.11290)

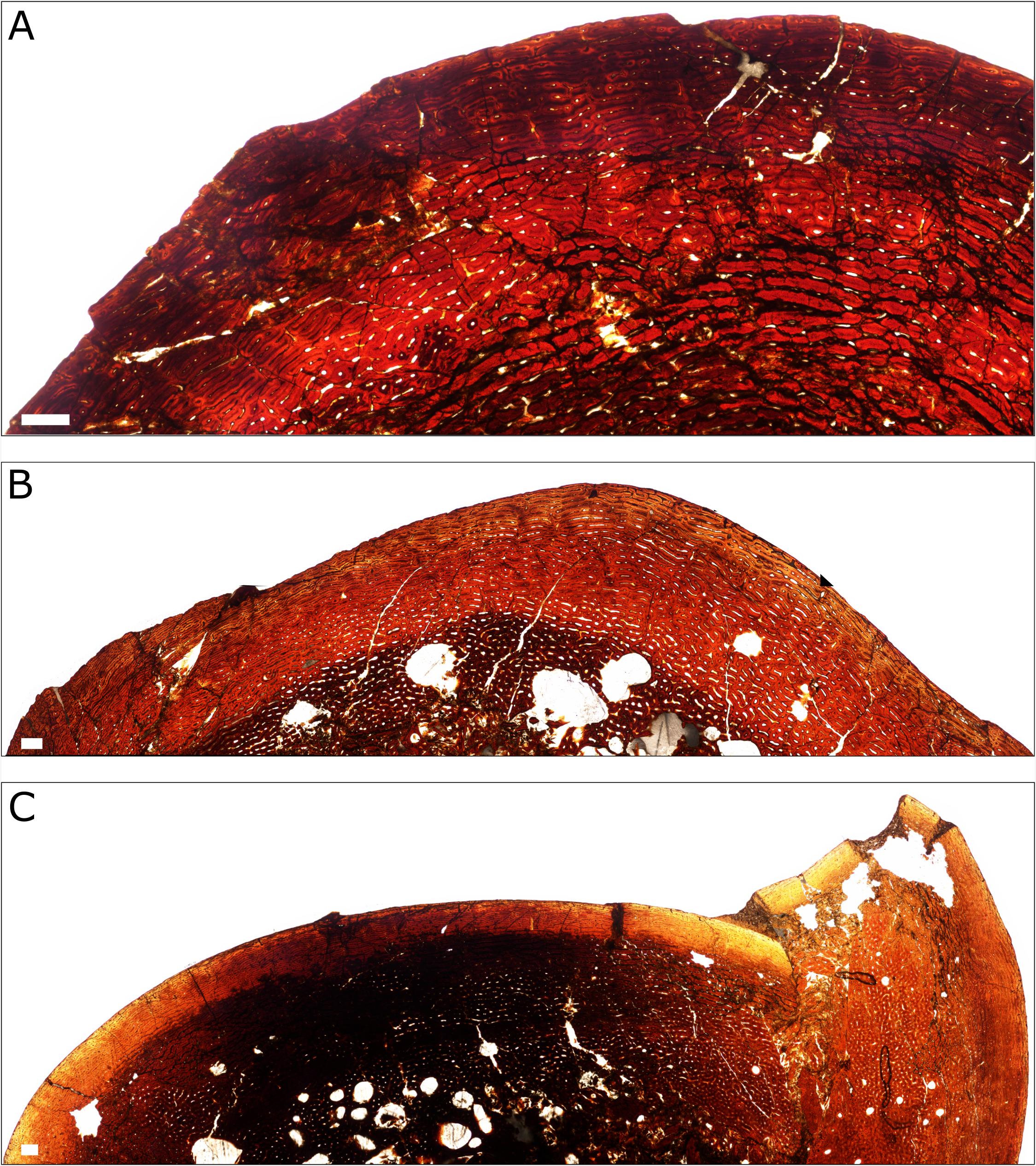

Supplement: Supplemental Information 1 — (A) UALVP 60534. (B) UALVP 60537. (C) UALVP 60536. Scale bars represent 500 µm. [file peerj-09-11290-s001.png]

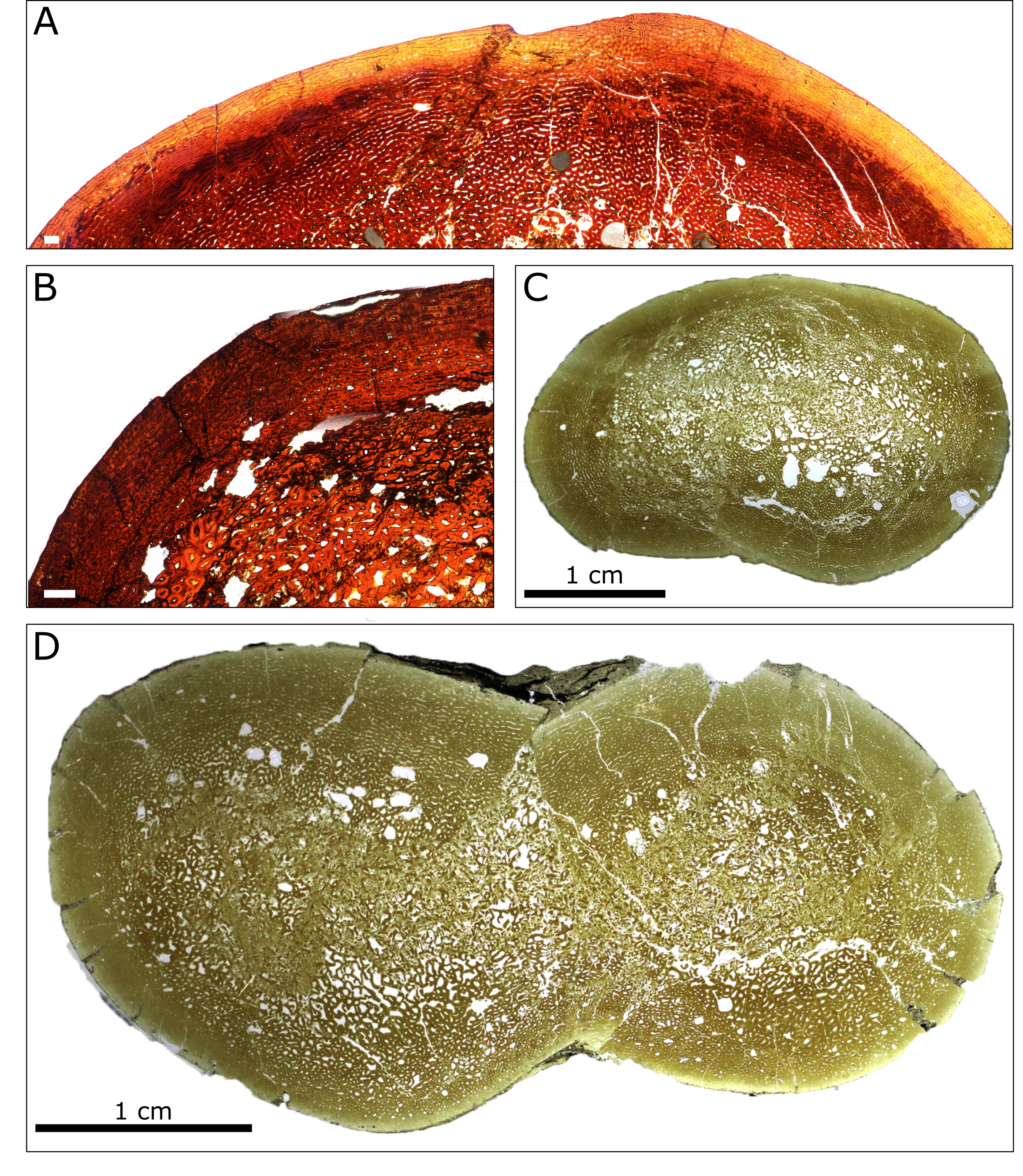

Supplement: Supplemental Information 2 — (A) UALVP 60533. (B) UALVP 60532. (C) TMP 1988.94.0006. (D) TMP 1991.137.0009. White scale bars in (A) and (B) represent 500 µm. Images (C) and (D) were provided by Russell Bicknell. [file peerj-09-11290-s002.png]

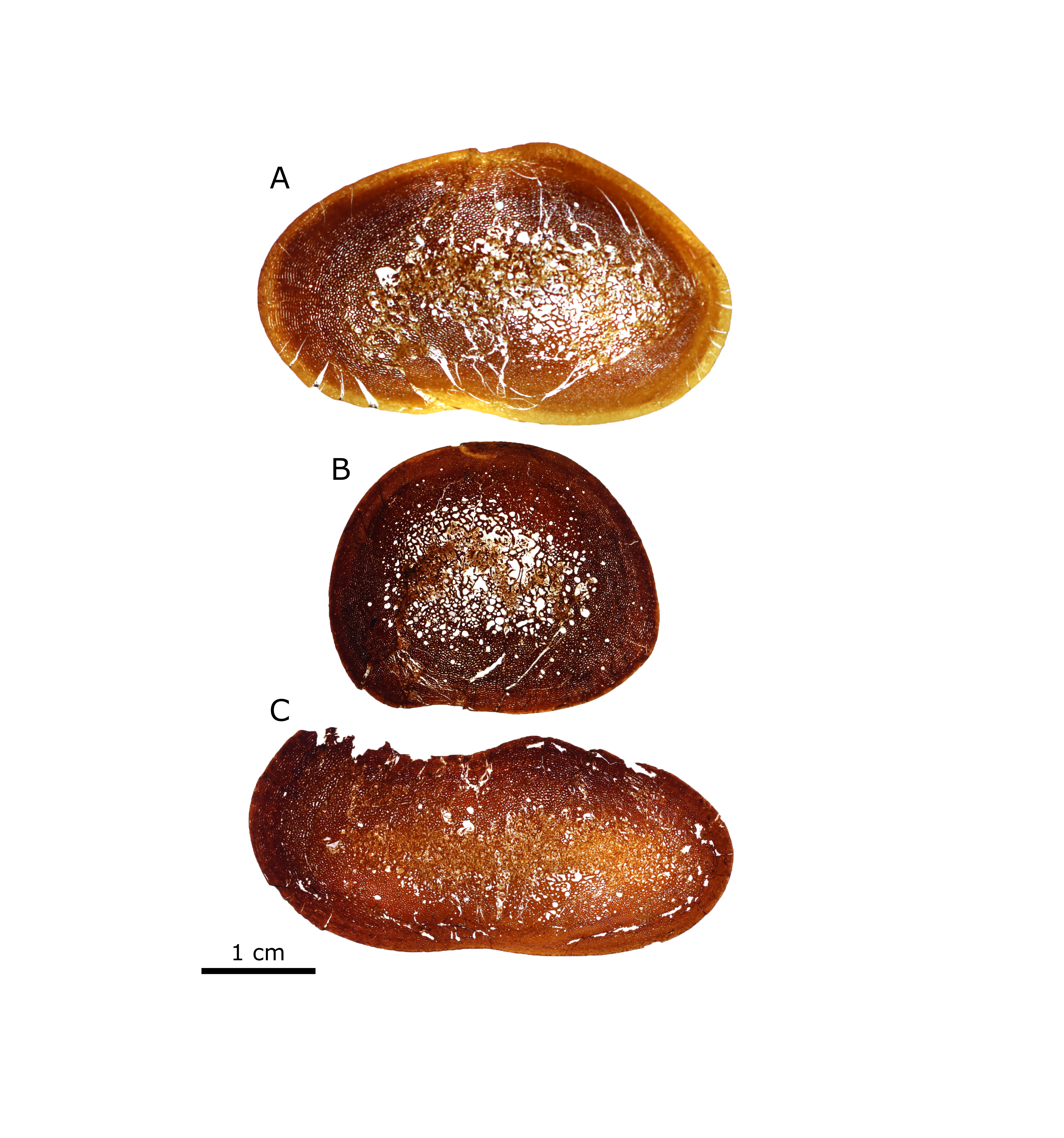

Supplement: Supplemental Information 3 — (A) UALVP 60533. (B) UALVP 60539. (C) UALVP 60532. [file peerj-09-11290-s003.png]
